# A role for GLUT3 in glioblastoma cell invasion that is not recapitulated by GLUT1

**DOI:** 10.1080/19336918.2021.1903684

**Published:** 2021-04-12

**Authors:** Catherine J Libby, Sajina Gc, Gloria A. Benavides, Jennifer L. Fisher, Sarah E. Williford, Sixue Zhang, Anh Nhat Tran, Emily R. Gordon, Amber B. Jones, Kaysaw Tuy, William Flavahan, Juan Gordillo, Ashlee Long, Sara J. Cooper, Brittany N. Lasseigne, Corinne E. Augelli-Szafran, Victor Darley-Usmar, Anita B. Hjelmeland

**Affiliations:** a Department of Cell, Developmental and Integrative Biology, University of Alabama at Birmingham, Birmingham, AL, USA; b Mitochondria Medicine Laboratory, Department of Pathology, University of Alabama at Birmingham, Birmingham, AL, USA; c Chemistry Department, Drug Discovery Division, Southern Research, Birmingham, AL, USA; d Department of Neurosurgery, Northwestern University, Chicago, IL, USA; e HudsonAlpha Institute for Biotechnology, Huntsville, AL, USA; f Department of Molecular, Cell and Cancer Biology, University of Massachusetts Medical School, Worchester, MA, USA; g Department of Genetics, University of Alabama at Birmingham, Birmingham, AL, USA; h O’Neal Comprehensive Cancer Center, University of Alabama at Birmingham, Birmingham, AL, USA; i Hugh Kaul Precision Medicine Institute, University of Alabama at Birmingham, Birmingham, AL, USA; j The Center for Clinical and Translational Science, University of Alabama at Birmingham, Birmingham, AL, USA; k UAB IMPACT Fund, University of Alabama at Birmingham, Birmingham, AL, USA

**Keywords:** Glucose transporter, glioblastoma, invasion, metabolism

## Abstract

The multifaceted roles of metabolism in invasion have been investigated across many cancers. The brain tumor glioblastoma (GBM) is a highly invasive and metabolically plastic tumor with an inevitable recurrence. The neuronal glucose transporter 3 (GLUT3) was previously reported to correlate with poor glioma patient survival and be upregulated in GBM cells to promote therapeutic resistance and survival under restricted glucose conditions. It has been suggested that the increased glucose uptake mediated by GLUT3 elevation promotes survival of circulating tumor cells to facilitate metastasis. Here we suggest a more direct role for GLUT3 in promoting invasion that is not dependent upon changes in cell survival or metabolism. Analysis of glioma datasets demonstrated that GLUT3, but not GLUT1, expression was elevated in invasive disease. In human xenograft derived GBM cells, GLUT3, but not GLUT1, elevation significantly increased invasion in transwell assays, but not growth or migration. Further, there were no changes in glycolytic metabolism that correlated with invasive phenotypes. We identified the GLUT3 C-terminus as mediating invasion: substituting the C-terminus of GLUT1 for that of GLUT3 reduced invasion. RNA-seq analysis indicated changes in extracellular matrix organization in GLUT3 overexpressing cells, including upregulation of osteopontin. Together, our data suggest a role for GLUT3 in increasing tumor cell invasion that is not recapitulated by GLUT1, is separate from its role in metabolism and survival as a glucose transporter, and is likely broadly applicable since GLUT3 expression correlates with metastasis in many solid tumors.

## Introduction

Altered metabolism is a hallmark of cancer [1] and has been implicated in the regulation of cancer invasion and metastasis [2–6]. One highly invasive and metabolically plastic tumor type is glioblastoma (GBM). Glioblastoma is the most common primary malignant adult brain tumor with a propensity to invade into the surrounding brain [7–10]. The invasive nature of this disease leads to recurrence, which is often within centimeters of the original tumor site, and ultimately death in nearly all patients [5,9,11,12]. The invasive nature of GBM makes it an incredibly difficult tumor to treat: these tumor cells invading normal brain cannot be removed by surgical resection and do not readily respond to current therapies. Improved understanding of the mechanisms of invasion and metabolism, as well as the links between them, in tumor growth, maintenance, and spread is likely to lead to novel treatments that will ultimately extend patient life expectancy.

The glycolytic shift of tumor cells is known to involve the SLC2A family of glucose transporters (GLUT) [13–16]. In the brain, glucose transporter 3 (GLUT3) is recognized as the neuronal glucose transporter, and glucose transporter 1 (GLUT1), is important for glucose uptake in astrocytes and the transport of glucose across the blood brain barrier [14,17–19]. GLUT1 is also ubiquitously expressed throughout the body. Differences in tissue expression between GLUT3 and GLUT1 may correlate with differential requirements for energy: GLUT3 has a five-fold higher affinity for glucose compared to GLUT1, allowing for preferential glucose uptake in environments with lower glucose concentrations [14,20]. Indeed, elevation of GLUT3 in subsets of less differentiated, highly metabolically plastic GBM cells called brain tumor initiating cells (BTICs) promotes survival in restricted glucose [2,21]. BTICs have also previously been reported to be more invasive than more differentiated cells within GBM tumors [22,23]. GLUT3 expression was also elevated in bevacizumab-resistant cells [24], and bevacizumab resistance is associated with a shift in metabolism, as well as a more invasive and mesenchymal-like phenotype [5,25–28]. However, a direct role for GLUT3 in modulating GBM invasion has not yet been reported.

Epithelial to mesenchymal transition and metastatic disease has been correlated with GLUT3 expression in other solid tumors such as those of the breast [29,30], liver [31], and lung [32]. Recently published papers demonstrated that GLUT3 was elevated in circulating tumor cells that have a propensity to target the brain [29] and that GLUT3 was necessary for their survival within the brain [30]. Further reports have linked GLUT3-YAP signaling to colon cancer metastasis [33], a pathway that is of conserved importance in some subsets of GBM cells [34]. Importantly, the data suggest that glycolytic shift/elevated glucose uptake mediated by GLUT3 increase circulating tumor cell survival which in turn promotes metastasis. High GLUT3 expression positively correlated with an increased incidence of metastasis in breast and head and neck cancers [35]. Furthermore, lower levels of GLUT3 correlated with a longer duration of metastasis-free survival in breast and head and neck cancers [36]. Through the use of publicly available data sets, we also observed a significant increase in GLUT3 expression in metastatic ovarian, head and neck, and colon cancers compared to primary tumors (Supplementary Figure 1) [37,38]. While these data suggest a role for GLUT3 in invasion and metastasis, a direct role for GLUT3 in invasion independent of pro-survival effects has yet to be investigated. Through our study, we have determined that GLUT3 has a role in mediating glioma invasion, outside of its role in metabolism, that is mediated by the C-terminal end of the protein.

**Figure 1.**
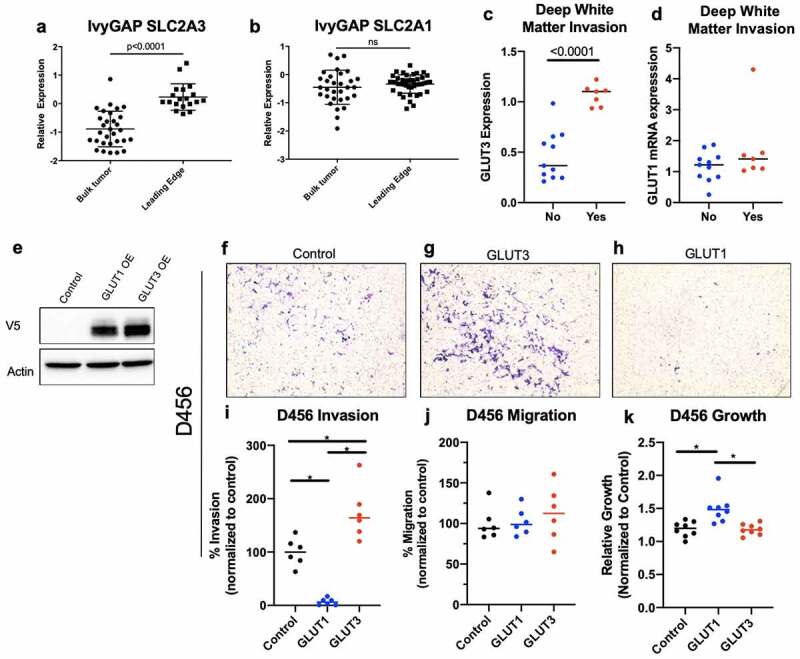
Increased GLUT3 expression correlates with increased GBM invasion in 0456 GBM cells

Analysis of lvyGap (a,b) and Rembrandt (c,d) data indicates that GLUT3 (a.c), but not GLUT1 (b.d) mRNA is significantly elevated at the GBM leading invasive edge. (e) Western blot of 0456 GBM expressing control, GLUT1-V5 cDNA, or GLUT3-V5 cDNA lentivirus plasmids with analysis for VS and actin levels. (f-k) 0456 GBM cells were incubated under low glucose conditions 16 hours prior to plating in invasion or migration Boyden chamber assays under low glucose and growth factor starvation with chemotaxis toward high glucose and growth factors. Representative images of invasion inserts at 10x magnification for 0456 (f-h} that were quantified using lmageJ (i). Migration was assessed using non-coated inserts and quantified using lmageJ (k) Analysis of 0456 growth under low glucose conditions for 36 hours, a time course similar to the total time of invasion assays. Data are average of the sum of six images per insert from three experiment for invasion and migration (*n* = 2). Growth results are from three experiment *n* = 3, ± sd, one-way ANOVA and Tukey’s multiple comparison test (•, *p* < 0.05).

## Results

### GLUT3 is elevated in invasive GBM cells

We previously reported that GLUT3 expression is correlated with worse glioma prognosis and is elevated in BTICs allowing them to preferentially survive in the low nutrient microenvironments commonly present in GBM tumors [21]. In order to further understand the relevance of elevated GLUT3 in GBM biology, we analyzed the Ivy Glioblastoma Atlas Project database and found GLUT3 (SLC2A3), but not GLUT1 (SLC2A1), expression was significantly elevated at the leading edge of GBMs (Figure 1 a,b) [35]. A correlation between elevated GLUT3, but not GLUT1, expression and white matter tract invasion was also noted in VASARI REMBRANDT data (Figure 1 c,d) [36,39]. This opens the possibility that invasion mediated by GLUT3 could contribute to the poorer patient prognosis associated with elevated GLUT3 expression.

### Elevated GLUT3 expression promotes GBM invasion in vitro

**Figure 2.**
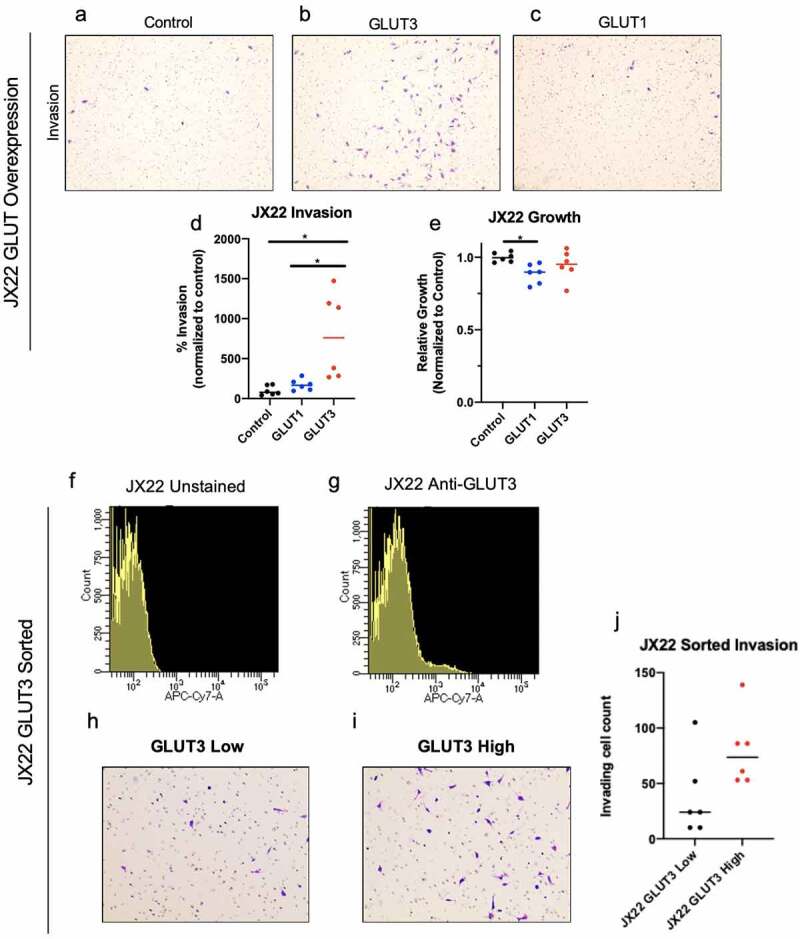
JX22 cells with elevated GLUT3 expression display increased invasion. (a-f) Representative images of JX22 GBM cell invasion inserts at 1Ox magnification exogenous GLUT overexpression (a-c) that were quantified using lmageJ (d). (e) Analysis of 0456 growth under low glucose conditions for 36 hours indicate no significant differences over a time course similar to the invasion assay. Histograms of fluorescent signal for unstained (f) or anti-GLUT3-Alexa Fluor 647 (g) JX22 cells from fluorescence-activated cell sorting. Representative images of invasion inserts for low (h) and high (i) GLUT3 expressing JX22 cells that were quantified using lmageJ. Data are average of the sum of six images per insert from two experiment for invasion (*n* = 3) and for growth results are from two experiments (*n* = 3),± sd, one-way ANOVA and Tukey’s multiple comparison test (*, *p* < 0.05)

To identify whether GLUT3 has a distinct role in cellular invasion, we overexpressed GLUT1-V5 or GLUT3-V5 cDNA using lentiviral infection in D456 and JX22 GBM patient derived xenograft (PDX) and U251 GBM cells. Expression of GLUT1-V5 or GLUT3-V5 was confirmed via qRT-PCR or western blot analysis after selection with Blasticidin S (Figure 1e, Supplementary Figure 2a). Overexpression levels of GLUT1 and GLUT3 were kept comparable to one another to limit potential dosing artifacts due to the overexpression system. To assess invasive capacity of GLUT3 or GLUT1 overexpressing cells, we utilized Boyden chamber assays with both glucose and growth-factors as chemo-attractants in the bottom well (Figure 1 f-i, Figure 2a-d,b-e). GLUT3 overexpressing cells, but not GLUT1 overexpressing cells, had significantly increased invasion compared to vector control cells in the Boyden chamber-based cell invasion assay (Figure 1f-i, Figure 2a-d, Supplemental Figure 2b-e). In D456 cells overexpressing GLUT3, invasive cell counts were elevated by at least 50% of controls (Figure 1i). Interestingly, we observed no significant differences in D456 cells in the Boyden chamber-based cell migration assay (Figure 1j). However, we did see an elevation in growth in GLUT1 overexpressing D456 cells indicating the potential for a ‘go versus grow’ phenomenon occurring in these cells (Figure 1k). These experiments were repeated in U251 cells where we obtained similar results in the invasion and migration assays. In U251 cells, GLUT3 overexpression increased invasion by 75% (Supplemental Figure 2b-e), but again we saw no differences in migration experiments (Supplemental Figure 2 f). In U251 cells, there were no differences in growth after 36 hours between the control, GLUT1 overexpressing, and GLUT3 overexpressing cells (Supplemental Figure 2g).

As U251 cells are a standard GBM cell line and the D456 PDX has a proneural subtype gene signature, we also utilized PDX cells of a mesenchymal subtype, JX22, for further investigation of the role of GLUT3 in invasion. Invasion was elevated by approximately 200% in JX22 cells overexpressing GLUT3 (Figure 2a-d) and no differences in growth were observed (Figure 2e). The lack of significant growth elevation in GLUT3 overexpressing cells indicates the observed invasion phenotype is not due to a proliferation advantage in these cells. In order to assess GLUT3 mediated invasion at physiologically relevant levels of expression that would be seen in tumors, we sorted for the GLUT3^high^ population from JX22 GBM PDX cells and observed GLUT3^high^ cells also trended to be more invasive than GLUT3^low^ cells (Figure 2f-j).

### The intracellular C-terminal tail of GLUT3 is necessary to induce GLUT3-mediated invasion

**Figure 3.**
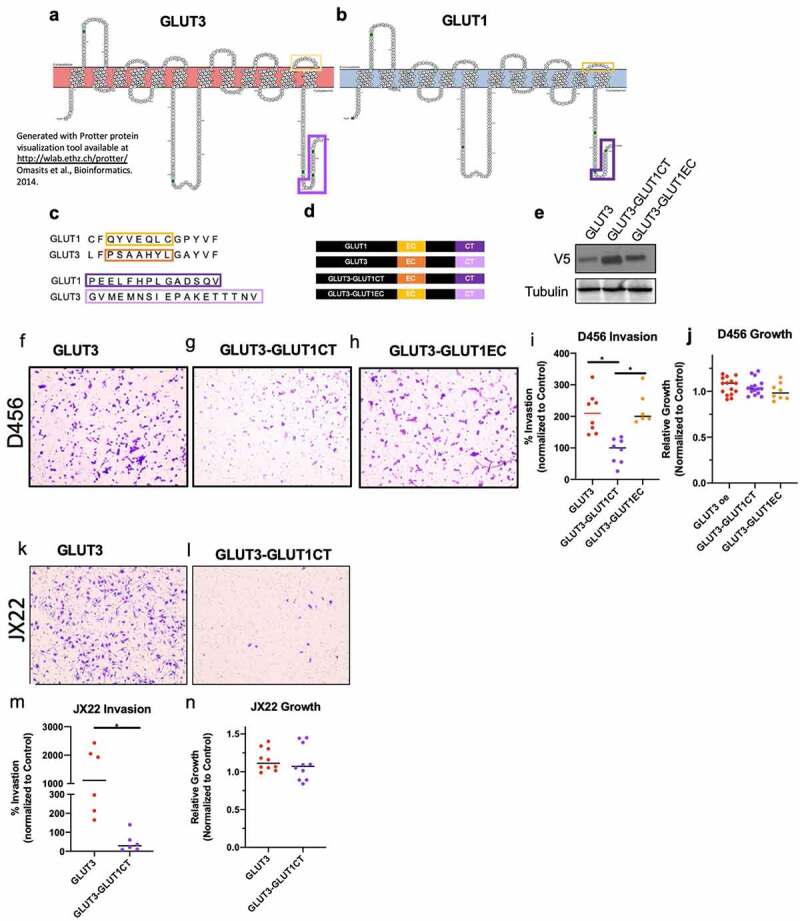
**The intracellular C-terminus, but not extracellular loop 6 is critical for GLUT3 mediated invasion**. Protein scheme of GLUT3 (a) and GLUT1 (b) highlighting regions of non-homology. (c) Sequence alignment of GLUT1 and GLUT3 protein sequences indicating regions of non-homology. (d) Schematic of GLUT3, GLUT1, and GLUT3 chimera proteins generated with immunoblotting in (e) demonstrating expression. (f h) Representative images of invasion chamber assays at 1Ox magnification with 0456 cells expressing indicated WT or chimeric proteins that were quantified using lmageJ (i). Analysis of 0456 growth under low glucose conditions for 36> hours indicate no significant differences over a time course similar to the invasion assay. (k, j) Representative images of JX22 cells expressing GLUT3 WT or GLUT3-GLUT1CT chimeric proteins Invasion assay inserts at 10x magnification which are quantified in (m). (n) Representative growth analysis of JX22 GLUT3 WT verses GLUT3-GLUT1CT chimeric protein expressing cells. Data are average of the sum of six images per insert from at least two experiments for invasion (*n* = 2–3) and for growth results are from three experiments (*n* = 3 or 4), ± sd, one-way ANOVA and Tukey’s multiple comparison test (*, *p* < 0.05)

**Figure 4.**
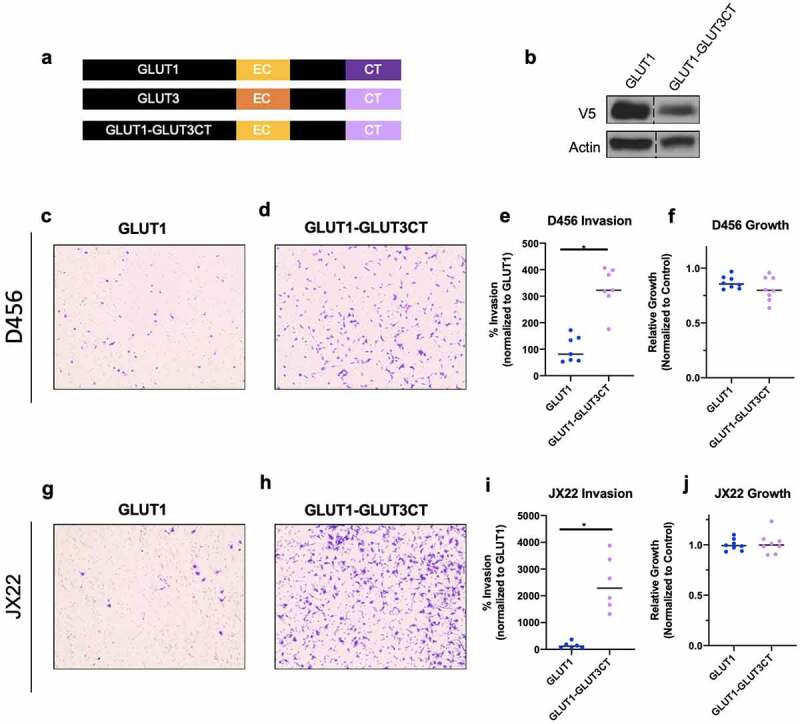
The intracellular C-terminus is sufficient to induce invasion in GLUT1 expressing cells. (a) Schematic of GLUT1 and GLUT1 chimera proteins generated with immunoblotting in (b) demonstrating expression. (c,d) Representative images of invasion chamber assays at 10x magnification with D456 cells expressing indicated WT or chimeric proteins that were quantified using lmageJ (e). (j) Determination of D456 growth under low glucose conditions for 36 hours (k,j). Representative images of JX22 cells expressing GLUT1 WT or GLUT1-GLUT3CT chimeric protein invasion assay inserts at 10x magnification which are quantified in (m). (n) 36 hour growth analysis of JX22 GLUT1 WT verses GLUT1-GLUT3CT chimeric protein expressing cells. Data are average of the sum of six images per Insert from three experiments (*n* = 2 or 3) for Invasion and for growth results are from two experiments (*n* = 4). ± sd, one-way ANOVA and Tukey’s multiple comparison test (*, *p* < 0.05)

The GLUT isoforms are highly homologous proteins, with GLUT1 and GLUT3 having nearly 80% sequence similarity. Comparison of GLUT1 and GLUT3 protein sequences identified two regions of non-homology near one another; one extracellular (EC) and one intracellular (the C-terminal end [CT]) (Figure 3a-c). The extracellular region is predicted to facilitate glucose recognition and binding and the intracellular region likely to facilitate protein–protein interactions. To determine if these regions may play a role in the invasive phenotype we observed, we generated chimeric proteins by swapping the GLUT1 sequence into the respective site in GLUT3 as to avoid mutations that are unstable and/or misfolded (Figure 3d). Previously, Inukai et al. utilized a similar strategy to assess apical versus basal membrane localization of GLUT1 and GLUT3 indicating that these chimeras should traffic to the membrane and be functional [40]. Following confirmation of successful expression of wildtype and chimeric proteins (Figure 3e), we assessed their invasive capacity. GLUT3-GLUT1EC had no effect on GLUT3 mediated glioma cell invasion, with cell quantification levels comparable to that of GLUT3 wildtype in D456 cells (Figure 3f,h,i). However, GLUT3-GLUT1CT attenuated glioma cell invasion, reducing invasion by over 60% compared to wildtype GLUT3 (Figure 3f,g,i). Additionally, these chimeric proteins did not alter growth in D456 cells (Figure 3 j). These results were recapitulated with JX22 (Figure 3k-n) and U251 cells (Supplementary Figure 2 h-k), suggesting the potential for this finding to be broadly applicable as these cells span two subtypes of GBM. We then performed the reciprocal experiments, utilizing a GLUT1-GLUT3CT chimeric protein (Figure 4a,b). Substitution of the GLUT1 C-terminus for that of GLUT3 elevated invasion in Boyden chamber assays compared to GLUT1 WT expressing cells, but again did not significantly alter cell growth (Figure 4 c-j). These data indicate that the C-terminal region of GLUT3 is responsible for mediating the pro-invasive phenotype observed here (Figures 3 and 4). Metabolic flux assays indicated no consistent metabolic changes that were associated with the invasive phenotype (Supplemental Figure 3). GLUT overexpression did not lead to significant changes in the rate of glycolysis indicating that glucose uptake is not a limiting factor for the cell’s glycolytic capacity. The minimal shifts in glycolytic metabolism indicate that the invasive phenotype is independent of glycolytic metabolism.

### Extracellular matrix organization gene signature is altered in GLUT3 overexpressing D456 GBM cells

**Figure 5.**
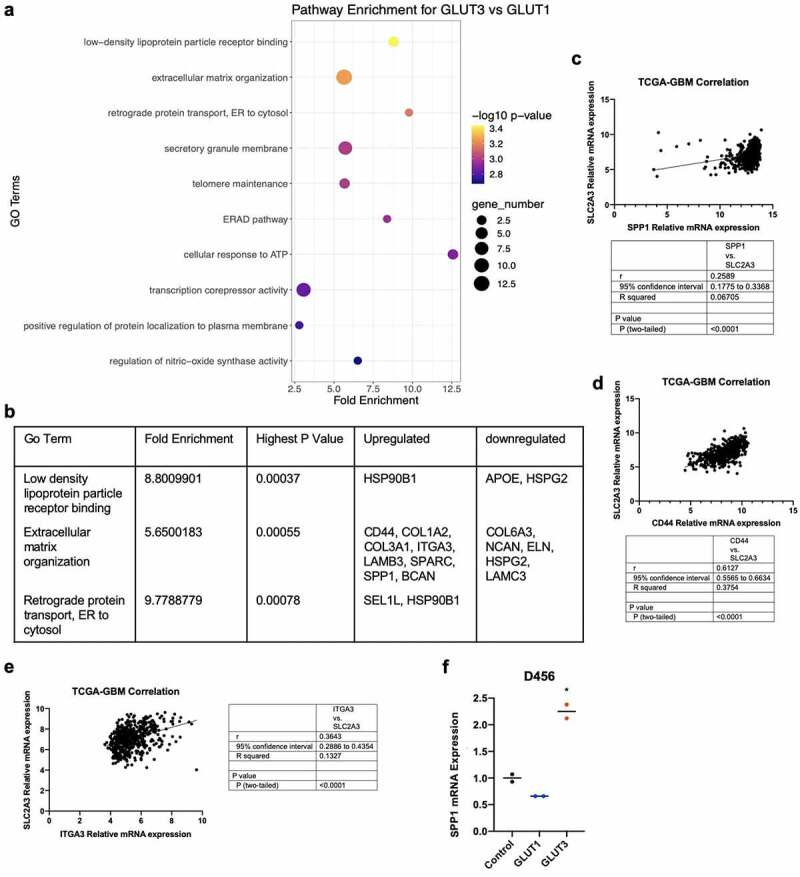
Transcriptomic analysis indicates differential regulation of extracellular matrix organization. (a) Dot plot of RNAseq data using the PathfindR package for pathway analysis to identify gene ontology pathway enrichment. (b) Top two enriched GO Terms with their enrichment scores. *p-*values, and differentially regulated genes. Co-expression of SPP1 (c}, CD44 (d), and ITGA3 (e) mRNA with SLC2A3 mRNA in the TCGA-GBM dataset. Statistical analysis performed using Pearson’s correlation. (d) qRT-PCR of SPP1 in control, GLUT1 overexpressing and GLUT3 overexpresslng 0456 cells (*n* = 2), one-way ANOVA and Tukey’s multiple comparison test (*, *p* < 0.05)

To begin understanding the molecular differences between GLUT3 and GLUT1 effects that could contribute to invasion, we performed RNA-seq analysis on control, GLUT1 overexpressing and GLUT3 overexpressing D456 GBM cells. We used PathFindR tool to identify gene ontology (GO) terms enriched among genes dysregulated in GLUT3 compared to GLUT1 overexpressing cells. The top three enriched GO terms in GLUT3 compared to GLUT1 overexpressing cells are low-density lipoprotein particle receptor binding, extracellular matrix organization, and retrograde protein transport (endoplasmic reticulum to cytosol) (Figure 5a, Supplemental Figure 4). From this analysis, we noted that multiple elevated transcripts within the extracellular matrix organization GO term, particularly, CD44, osteopontin (SPP1), SPARC, and integrin A3 (ITGA3) (Figure 5b), have been widely studied in GBM [41–48] and implicated with cancer invasion/metastasis and severity [41–48]. Evaluating the correlation between these genes using The Cancer Genome Atlas (TCGA), we determined that there is a positive correlation between SLC2A3 (GLUT3) and SPP1 (OPN) (Figure 5c), in addition to CD44 (Figure 5d), and ITGA3 (Figure 5e). The upregulation of SPP1 was validated via qRT-PCR, in which there is an over twofold upregulation of SPP1 mRNA expression in GLUT3 overexpressing D456 cells (Figure 5f). However, we did not see consistent upregulation of several other genes noted in the extracellular matrix organization GO term from the RNA-seq analysis (data not shown).

## Discussion

Previous work has indicated a correlation between GLUT3 and cancer metastasis [29–33] that is thought to depend on the ability of GLUT3 to promote circulating tumor cell survival. However, our results indicate a direct and distinct functional role for GLUT3 in mediating cancer cell invasion that has of yet not been reported to our knowledge. This invasion phenotype was independent of changes to growth or migration, highlighting that this is not a survival advantage due to GLUT3 overexpression. Interestingly, in the D456 cells GLUT1 significantly reduced invasion while also increasing cell growth (Figure 1h,i,k). This could indicate a ‘go versus grow’ phenotype in these cells; however, this was not a broad phenomenon. There may be a subpopulation of cells with a reliance on GLUT1 for sustained proliferation. Alternatively, in U251 cells GLUT1 overexpression resulted in a slight decrease in invasion and growth (Supplemental Figure 2d,e,g) and in JX22 cells there was a nonsignificant minor elevation in invasion, but a decrease in cell growth (Figure 2c,d,e). It is possible that GLUT1 interacts with different protein than GLUT3 and that this influences cellular signaling in ways not yet fully understood. GLUT1 may interact with other proteins to actively inhibit invasion and promote other cellular processes. The lack of invasion associated with high GLUT1 expression may also account for the lack of survival differences between GLUT1 high and low expressing tumors. Very little is known regarding differential functions between the GLUT family members beyond sugar transport and this is an interesting area for future studies.

We show that the pro-invasive phenotype in GLUT3 overexpressing cells is due to its C-terminus (Figures 3 and 4). Chimeric GLUT3 in which the C-terminus was substituted with that of GLUT1 abrogated invasion (Figure 3). In converse experiments, transferring the GLUT3 C-terminal sequence into GLUT1 increased invasion (Figure 4). The overexpression of GLUT1 or GLUT3 wild type or chimeric proteins had minimal effects on glycolytic metabolic flux that did not correlate with invasive phenotype, thus indicating that the invasive phenotype is independent of glycolytic metabolism. Further studies would be required to identify alternative metabolic pathways that may be activated with GLUT3 elevation, such as changes in lipid metabolism as suggested by the RNAseq data (Figure 5). However, we believe that these differential functions may contribute to the need for multiple isoforms of GLUTs in the body, each expressed in particular tissues [13–16,49,50]. This is also highlighted by the presence of a specific GLUT1 C-terminal binding protein (GLUT1CBP) that binds a PDZ domain within the C-terminus of GLUT1 which facilitates interactions with the cytoskeleton [50]. The region with which the GLUT1CBP is known to interact is not identical to the regions we replaced in our chimera proteins, indicating the potential for multiple regulatory regions within the intracellular C-terminal tail of the GLUT proteins. The report of specific interactions with the GLUT1CT indicates that this may also be true for GLUT3; however, we know of only one study to date specifically assessing differential functions of this region in GLUT3 [40]. The study by Inukai *et al*. generated GLUT1/GLUT3 chimeric proteins where 13 to 290 amino acids from the C-terminus of GLUT3 were transferred into GLUT1 [40]. The chimeric protein containing at least the final 19 amino acids of the GLUT3 c-terminus showed increased localization of GLUT1/GLUT3 chimeric protein to the apical membrane rather than the basal membrane in canine kidney epithelial cells [40]. These data suggest that there can be a specific subcellular localization for GLUT3 that contributes to its function within the cell. Interestingly, the GLUT3 C-terminus shares some homology with regions of proteins that are reported in the literature to play a role in invasion such as TNC, CD40, SPIRE1, GPR98, and PNN [51–57]. These similarities are not shared by GLUT1 indicating that there may be novel protein interactions with GLUT3 that promote invasion. Indeed, it has been reported that GLUT3 is important for trophoblast invasion for successful implantation of embryos during development [58]. Additionally, many metabolic enzymes have been reported to have functions independent of a direct role in metabolism [59–61]. For example, PKM2 is able to phosphorylate histone H3 and SREBPs in addition to its function in glycolysis [59]. Our results widen this to other proteins involved in metabolism, as GLUT3 currently has no known direct function beyond hexose transport. We believe this is an area that is of great interest and that our studies could lead to a number of intriguing findings for the differential roles of the 14 GLUT family members.

The RNA-sequencing data provide several potential avenues for further investigation to understand the impact of GLUT3 on the extracellular matrix. SPP1, one of the genes upregulated in GLUT3 overexpressing cells (Figure 5), encodes the protein osteopontin (OPN), which has been widely studied in cancers, including glioma. OPN has been shown to mediate cell adhesion and to promote cancer invasion and metastasis [41,62,63]. OPN also interacts with CD44, another molecule shown to be upregulated in the GLUT3 overexpressing cells (Figure 5) [41,62,64]. CD44 is a hyaluronan receptor that has been implicated in regulating cell motility and adhesion to promote cancer metastasis [41,64]. Interestingly, CD44 is commonly associated with a more mesenchymal cell population posing the possibility that high GLUT3 expression induces a mesenchymal shift [65–68]. CD44 and OPN have also been linked to multiple other commonly dysregulated pathways in cancer including but not limited to WNT/beta-catenin and AKT which have also been shown to influence invasion [41,65,69]. While these data suggest that the regulation of OPN and CD44 expression by GLUT3 upregulation deserves to be studied in more depth, we recognize the limitation of these *in vitro* results. The normal brain and brain tumor extracellular matrix and cell–cell interactions are complex and the brain environment will impact cellular behaviors *in vivo*. Evaluation of the GLUT3 invasion phenotype in the context of brain extracellular matrix components including hyaluronan is critical as is evaluation in tumor-endothelial cell interactions considering GBM cells can move along existing blood vessels to invade normal brain [70]. Further analysis of the properties of such as morphology of cancer cells with GLUT3 elevation, including those of other tumor types, with different matrix substrates [71] is an important area of future investigation.

GLUT3 and GLUT1 are the most commonly elevated GLUTs in cancers, including GBM. Their high degree of homology made it incredibly interesting that GLUT3 has such a significant correlation with patient prognosis (both in terms of overall survival and metastatic free survival) that was often not mimicked by GLUT1. The fact that this neuronal glucose transporter is elevated in multiple cancers combined with the distinct invasive role described here in GBM suggests a broader role for GLUT3 in invasion and metastasis. GBM infiltration into the normal brain ultimately leads to tumor recurrence very near the tumor resection border and this recurrence leads to death in nearly all cases of the disease [5,7–12]. Due to the nature of the brain, excess tissue cannot be resected to expand tumor margins to remove more of the invasive cells. Therefore, drugs that are brain penetrant are highly attractive to improve patient survival. Understanding drivers of GBM invasion will be critical for furthering drug development to improve patient outcomes. Drugs to target GLUT3 could be better designed to inhibit molecular functions outside its role in metabolism as a means to limit potential brain toxicities, potentially by targeting the C-terminal tail or the protein interactions driving GLUT3 mediated invasion. Additionally, GLUT3 targeted therapies would be attractive for a number of other cancers that have an elevation of GLUT3 such as lung, liver, colon, head and neck, and breast cancers where high-grade survival remains poor.

## Methods

**Cells and GBM patient-derived xenografts**: GBM patient derived xenografts D456 and JX22 were obtained from Dr. Darrel Bigner at Duke University and Dr. Jann Sarkaria at Mayo Clinic. The xenolines used here have been assessed for mutation status and relative transcript levels to determine molecular subtypes. Xenografts were dissociated using papain (Worthington Biochemical Corporation) and propagated in vitro using DMEM/F12 basal media (Invitrogen) supplemented with epidermal growth factor (EGF), fibroblast growth factor, sodium pyruvate, penicillin/streptomycin, and the B27 equivalent GEM21 (Gemini Bio Products). U251 cells were obtained as a kind gift from Dr. Corinne Griguer. CSC293T cells were generated and propagated as previously described [72]. Low glucose media utilized in some experiments consists of a dilution of 1 mL of NeurobasalA (Gibco) in 9 mL of Neurobasal minus glucose (Gibco), without the addition of any supplements. Prior to plating for experiments, cells were split with Accutase (Gibco) and counted.

**Protter protein visualization for GLUT1 and GLUT3**: Using the Protter [73] online interphase GLUT1 and GLUT3 topology visualizations were generated using the UniProt accession codes GTR1_ Human and GTR3_Human, respectively. Minor stylistic changes were made to the standard generated image using the tools provided.

**Protein alignment**: Alignment of GLUT1 and GLUT3 sequences was performed using NCBI BlastP and the FASTA sequences for the Uniprot accession codes above.

**Gene overexpression**: CSC293T cells were transiently co-transfected with psPAX2, pCMV-VSVG and pLX304 lentiviral plasmids containing GLUT3 or GLUT1 (Genecopia) using FuGENE® HD Transfection Reagent (Promega) as previously reported [72,74,75]. Virus titer was determined using Lenti-X qRT-PCR Titration Kit (Takara). Addition of Blasticidin S to cell culture media was used to select cells with stable overexpression. pLX304 lentiviral plasmids were mutated to generate chimeric proteins by Bioinnovatis, Inc (Rockville, MD) and plasmids sequenced to verify the desired mutations were present.

**mRNA extraction, cDNA generation and qRT-PCR**: Total mRNA from cells after 16-hour incubation in low glucose media was harvested using TriZol (ThermoFisher) and synthesized into cDNA using the iScript™ cDNA Synthesis Kit (BioRad). qRT-PCR was performed on the generated cDNA with the SsoAdvanced™ Universal SYBR® Green Supermix (BioRad). The relative expression of GLUT1 and GLUT3 was measured using primer pairs that recognize GLUT1 or GLUT3 cDNA (GLUT1: ACAACCAGACATGGGTCCAC and GTTAACGAAAAGGCCCACAG) (GLUT3: AGCTCTCTGGGATCAATGCTGTGT and ATGGTGGCATAGATGGGCTCTTGA). Primers against SPP1 were presynthesized from BioRad for PrimePCR applications (qHsaCID0012060). The data were analyzed and normalized against ACTB (AGAAAATCTGGCACCACACC and AGAGGCGTACAGGGATAGCA) expression to determine relative expression of target genes similar to prior reports [21,76].

**Western blotting and antibodies**: Cells were harvested following 16-hour incubation in low glucose media and lysed using M-PER (Thermo Scientific). Protein concentration was determined using the BCA assay (Thermo Scientific). Prior to electrophoresis on 4–20% Tris-Glycine Mini Gels (Invitrogen), protein lysates were denatured with Novex Tris-Glycine SDS sample buffer and NuPAGE sample reducing agent (Invitrogen). Protein was then transferred to nitrocellulose membranes (BioRad) and blocked using Pierce Protein Free Blocking Buffer. Primary antibodies for Western blot: mouse anti-V5 (Invitrogen) and Rabbit anti-Actin (Sigma). (n = 3)

**Measurement of cell growth**: Cell numbers were determined using crystal violet staining and solubilization in 10% acetic acid [77]. 1 × 10^4^ cells were seeded in 96-well plates coated with Geltrex (ThermoFisher) to promote cell adhesion and allowed to recover and attach overnight. The following day, the media was changed to low glucose media and incubated for 36 hours. At the end of experiments, cells were fixed in 10% formalin overnight at 4°C, stained with 0.2% crystal violet, and then washed x3 with diH_2_O to remove excess staining solution. Crystal violet was solubilized using 10% acetic acid and read at 590 nm using the Biotek synergy H1 microplate reader.

**Flow cytometry and cell sorting**: Cells from culture or directly isolated the previous day from subcutaneous GBM xenografts were used for flow cytometry. Cells were washed with cold DMEM:F12 (Gibco) and counted. Cells were resuspended in 90 uL of DMEM:F12 per 7 × 10^6^ cells and incubated with GLUT1 (BDBiosciences) or GLUT3 antibody (Invitrogen or R&D), corresponding IgG control, or viability dye for 30 minutes or 15 minutes, respectively. Cells were sorted with the assistance of the Flow Cytometry Core at the University of Alabama at Birmingham. The top and bottom 2–8% was collected for high versus low expression and used for experiments.

**Boyden chamber invasion and migration**: 5 × 10^6^ cells were plated on Geltrex (ThermoFisher) and allowed to recover. Media was then replaced low glucose media overnight. 0.8 µm pore Boyden chamber inserts coated with reduced growth factor Matrigel (Corning) (Invasion) or non-coated (Migration) were rehydrated for 2 hours prior to plating similar to prior reports [78,79]. 1 mL of DMEM/F12 basal media (Invitrogen) supplemented with epidermal growth factor (EGF), fibroblast growth factor (FGF), sodium pyruvate, penicillin/streptomycin, and the B27 equivalent GEM21 (Gemini Bio Product) was placed in each well of a 24 well plate to be used. 2.0 × 10^4^ cells were plated in low glucose media in the Boyden chambers following rehydration in triplicate. Cells were allowed to invade for 8–16 hours or migrate for 4–8 hours depending on cell type, and then fixed with 10% formalin overnight in 4°C. Cells were stained with crystal violet, and then the inserts were washed, cleaned with diH_2_O, and imaged. Six images covering each insert were totaled by hand counting and/or ImageJ particle analysis and graphed using GraphPad Prism. All experiments were performed in duplicate with at least two technical replicates.

**Glycolytic Stress Test**: A Seahorse XFe96 Analyzer (Agilent. Santa Clara, CA) was used to perform a Glycolysis Stress Test (GST) in D456 cells as prior described [80,81]. In Brief, D456 cells were seeded (20,000/well) into a XF96 cell culture microplate in complete BTIC medium and maintained in a 5% CO2 incubator at 37°C overnight prior to the experiments. The day of the assay, media in the plate with cells was then changed to assay media (Seahorse basic DMEM with 2 mM L-glutamine, 1 mM pyruvate at pH 7.4 and 37°C) and maintained in a non-CO2 incubator at 37 °C for 1 h prior to the assay. The GST was conducted by subsequent injections of glucose (10 mM final concentration), oligomycin (1 μg/mL final concentration), and 2-deoxyglucose (2-DG; 50 mM final concentration). Experiments were performed in triplicate with six technical replicates.

**RNA sequencing analysis**: 3 × 10^6^ cells were plated on Geltrex and allowed to recover overnight. Cells were pelleted following overnight incubation with low glucose media and frozen at −80°C until RNA extraction (n = 3 per cell type). RNA extraction was performed using the Norgen Total RNA extraction kit; (Norgen cat. # 37,500, 25,720). RNA quality numbers, RIN, were measured using BioAnalyzer (Agilent) and ranged from (9.7–10 RIN). We used 1000 ng of total RNA as input to the NEBNext Poly(A) mRNA Magnetic Isolation Module (NEB cat# E7490S). PolyA depleted RNA was used as input to the NEBNext Ultra RNA Library Prep Kit for Illumina (NEB cat# E7530S). Libraries were barcoded using the NEBNext Multiplex Oligos for Illumina (NEB cat# E7335S). We pooled all samples to achieve equal representation and sequenced on one lane of an Illumina HiSeq 2500, paired end with 50 base pairs and sequenced an average of 30.78 million reads per sample with an average mean quality score (PF) of 35.23. We processed the raw files using aRNA-pipe which implements STAR for alignment and HTSeq for the generation of count tables [82]. This approach is similar to that previously described in Boyd *et al*. [83]. The differential gene expression (DGE) and GO term enrichment analyses were analyzed in R (version 3.6.2) with RStudio (version 1.2.5033). The raw counts were variance stabilized with DESeq2’s (version 1.26.0) varianceStabilizingTransformation function [84]. These variance stabilized counts were used by the prcomp() function for Principal Component Analysis (PCA) analysis. The PCA scatterplot of PC1 and PC2 showed a batch effect (Supplemental Figure 5). The DESeq2 design formula for analyzing differentially expressed genes between GLUT1 and GLUT3 included the experimental groups and this batch effect. Other DESeq2 parameters were at default values. Genes with raw counts average lower than or equal to 10 were excluded in the DESeq2 analysis. Based on the DESeq2 results, genes with an adjusted p-value less than 0.05 and an absolute log_2_ fold change greater than 1.0 were considered significant. We converted Ensembl IDs to gene symbols with the R packages ENSDB.Hsapiens.v75 (version 2.99.0) and AnnotationDBI (version 1.48.0) [85,86]. Gene symbols with adjusted p-values and log_2_ fold change values from DESeq2 were used as input for the pathfindR R package (version 1.4.2) [87] to assess gene enrichment by Gene Ontology (GO) terms. We used default parameters for the run_pathfindR function with the exception of the gene_sets parameter which was set to ‘GO-ALL’. This analysis produced an enrichment chart of the top enriched GO terms and a table of all enriched GO terms. The RNA sequencing data are deposited in GEO: GSE148739.

**Data Set analysis**: SLC2A3 gene expression and tumor site data were downloaded from Oncomine [37,38] for Bittner Ovarian, Tsuji Colon and Cromer Head and Neck cancer datasets to assess expression in primary versus metastatic lesions. SLC2A3 expression data were downloaded from the IVYGAP [35] data to assess the expression of SLC2A3 in various histological regions. TCGA-GBM SPP1 and SLC2A3 gene expression data were downloaded from gliovis [88] and plotted to assess expression correlation. VASARI (Visually AceSAble Rembrandt Images) data were downloaded from The Cancer Imaging Archive (TCIA). Data consisted of scores from three radiologists on the full VASARI feature set, including deep white matter invasion (feature number f21). Data from 18 glioblastoma patients that also had REMBRANDT mRNA expression data (Affymetrix GeneChips) were analyzed for GLUT3 expression (probe 202499_s_at), GLUT1 expression (probe 201249_at) and the presence of deep white matter invasion as determined by at least one of the three VASARI radiologists.

## Supplementary Material

Supplemental Material

## Additional statistical analysis

All statistics not described as executed in R, were performed with GraphPad Prism Version 7 (GraphPad Software Inc., La Jolla, CA). One-way ANOVA and t-tests were performed with Dunnett’s or Tukey’s test for multiple comparisons with a confidence level of 95%. Correlation analysis was performed using Pearson’s correlation analysis with a confidence interval of 95%.
