# Supplementary figures and images for: A role for GLUT3 in glioblastoma cell invasion that is not recapitulated by GLUT1
Source: Cell Adh Migr. 2021 Apr 12;15(1):101–15. doi: 10.1080/19336918.2021.1903684 (PMC8043167; doi:10.1080/19336918.2021.1903684)

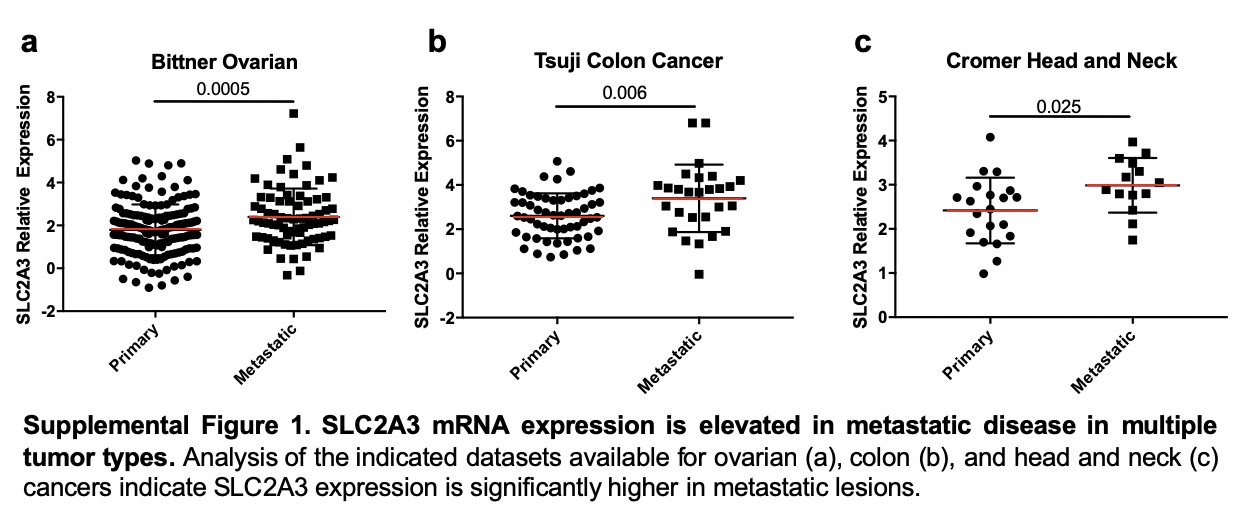


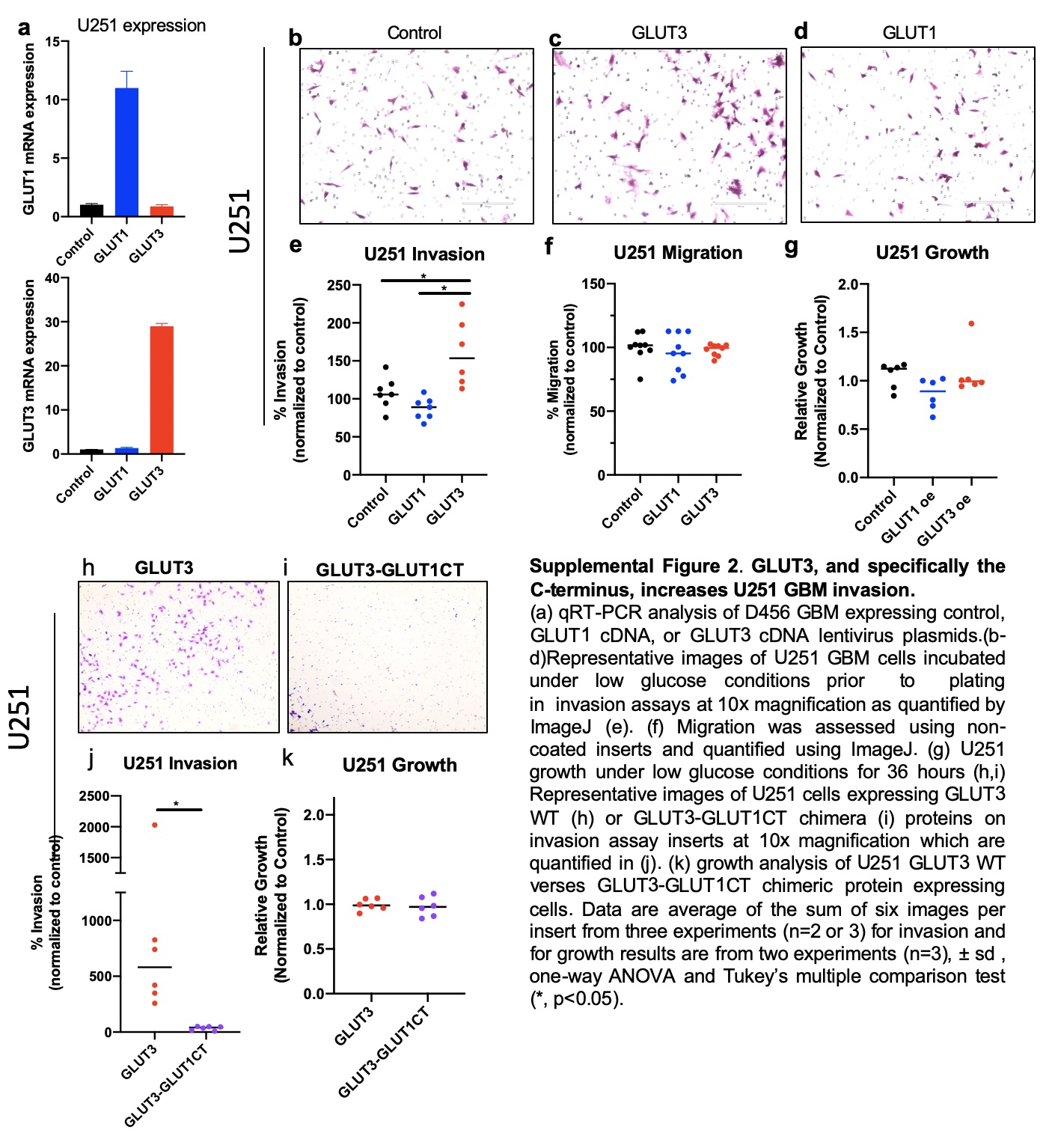


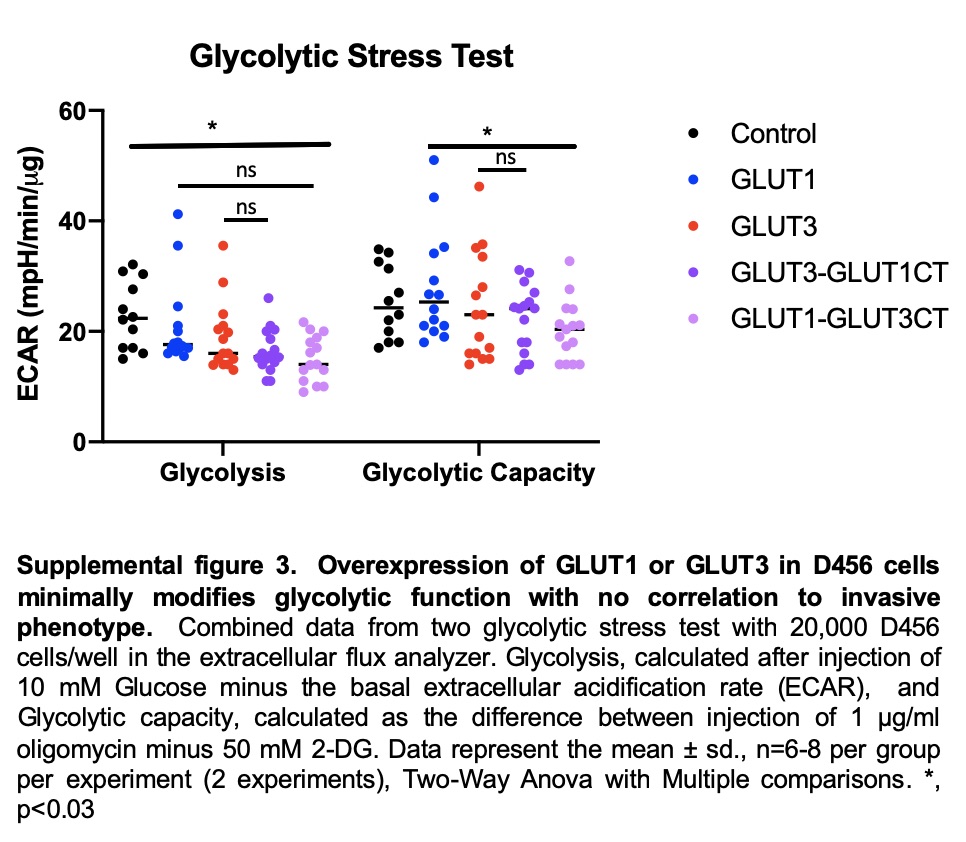


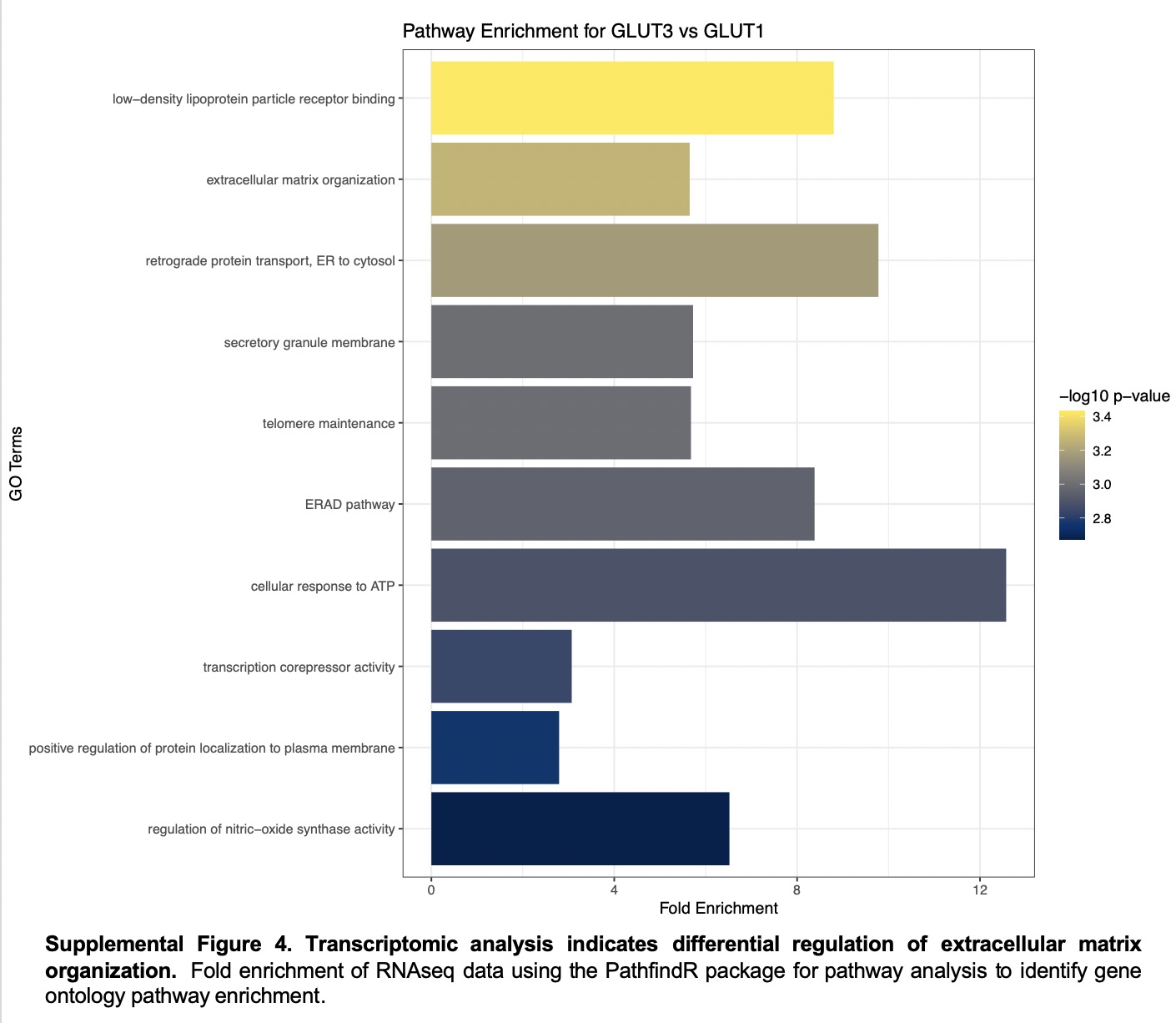


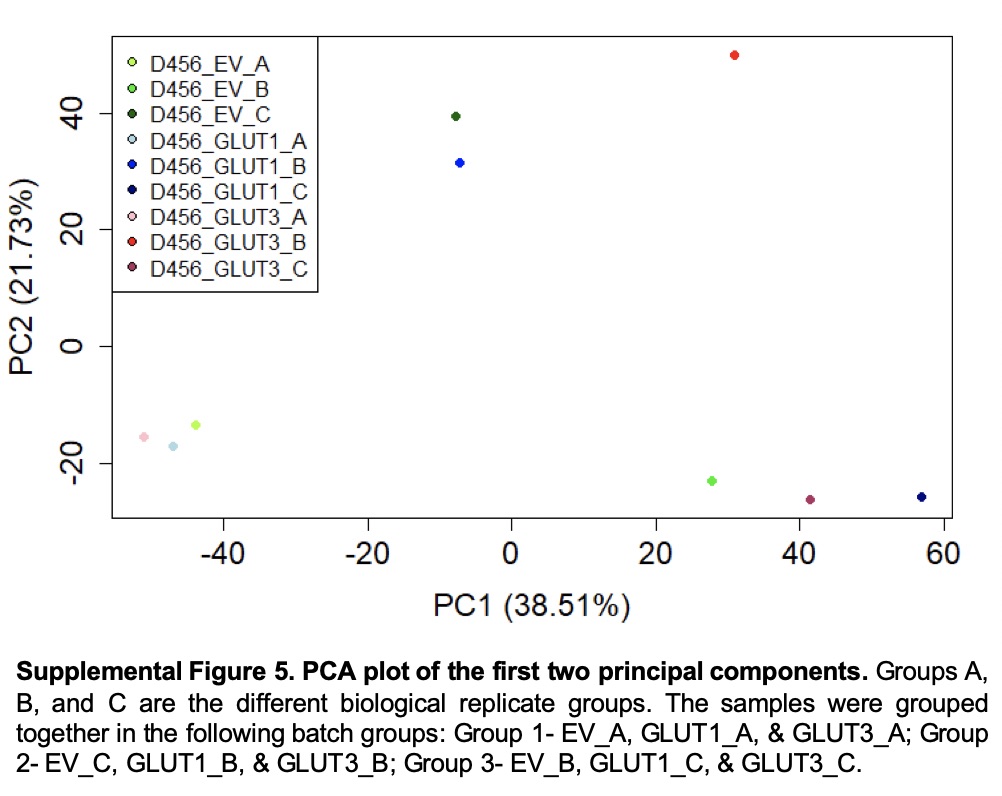

Supplement: Supplemental Material [file KCAM_A_1903684_SM9458.docx]
